# Intravitreal Dexamethasone Implant Switch after Anti-VEGF Treatment in Patients Affected by Retinal Vein Occlusion: A Review of the Literature

**DOI:** 10.3390/jcm13175006

**Published:** 2024-08-23

**Authors:** Livio Vitiello, Filippo Lixi, Alessia Coppola, Giulia Abbinante, Vincenzo Gagliardi, Giulio Salerno, Ilaria De Pascale, Alfonso Pellegrino, Giuseppe Giannaccare

**Affiliations:** 1 Eye Unit, “Luigi Curto” Hospital, Azienda Sanitaria Locale Salerno, 84035 Polla, SA, Italy; livio.vitiello@gmail.com (L.V.); alessiacoppola330@gmail.com (A.C.); giulia.abbinante@gmail.com (G.A.); v.gagliardi@aslsalerno.it (V.G.); giuliosalerno@hotmail.it (G.S.); idepascale@outlook.it (I.D.P.); al.pellegrino@aslsalerno.it (A.P.); 2 Eye Clinic, Department of Surgical Sciences, University of Cagliari, 09124 Cagliari, CA, Italy; f.lixi0106@gmail.com

**Keywords:** intravitreal dexamethasone implant, retinal vein occlusion, switch

## Abstract

Nowadays, retinal vein occlusion (RVO) is the second most prevalent cause of vision loss associated with retinal vascular disease. Intravitreal injections are currently known as a major advancement in ophthalmology, particularly in the treatment of RVO and other retinal disorders. Particularly, the first line of therapy is usually anti-vascular endothelial growth factor (VEGF) drugs. Notably, for RVO eyes that have not responded to anti-VEGF therapy, an intravitreal dexamethasone (DEX) implant 0.7 mg (Ozurdex^®^, AbbVie Inc., North Chicago, IL, USA) is considered a suitable therapeutical substitute. Actually, investigations carried out in the real world and clinical trials have shown the safety and the efficacy of intravitreal DEX implants for treating this retinal disease. For this reason, choosing patients carefully may thus be essential to reduce the number of injections that clinics and hospitals have to do to manage RVO and its complications. The primary aim of this review is to summarize the pathophysiology of this retinal vascular disease, as well as the clinical and ocular imaging features that may support a switch from prior anti-VEGF treatment to intravitreal DEX implant, to provide the RVO patients with the best possible treatment to ensure maximum visual recovery.

## 1. Introduction

Nowadays, the second most common cause of retinal-vascular-disease-related visual loss is retinal vein occlusion (RVO) [1,2,3]. RVO has historically been classified into two primary categories, notwithstanding the difficulty in clinical classification: (1) central RVO (CRVO), where the obstruction occurs within the central retinal vein located within the optic nerve and affects the entire retina, involving 0.1% to 0.4% of the global population, and (2) branch RVO (BRVO), which affects 0.6% to 1.2% of people worldwide and is characterized by an obstruction within one of the branches of the retinal vein, usually at the site where the retinal artery and vein cross [4,5].

There is ongoing discussion over the best ways to manage RVO and its complications, encompassing conservative (especially wait-and-see strategy, considering that CRVO could also improve spontaneously) [6,7], medical, and surgical methods, due to the incomplete characterization of various elements of the underlying etiology [8]. Furthermore, the diagnostic and treatment strategies for these vascular disorders continue to be expanded by new pharmaceutical choices and imaging devices [9,10,11,12,13].

Macular edema, which affects up to 15% of eyes with BRVO and 30% of eyes with CRVO, is the most frequent vision-threatening consequence of RVO [14,15]. For macular edema resulting from RVO, intravitreal anti-vascular endothelial growth factor (VEGF) therapy is considered the cornerstone of treatment [16]. In fact, macular edema and retinal ischemia can occur in different degrees as a result of chronic vascular obstruction. Pressure accumulation inside the clogged retinal veins might harm the vascular wall, resulting in vascular leakage and the development of macular edema. Thus, macular edema develops as a result of the release of VEGF and other inflammatory mediators brought on by the abrupt temporary retinal ischemia [17]. For this reason, three anti-VEGF drugs, aflibercept, ranibizumab, and bevacizumab, are frequently used to treat macular edema related to RVO [18,19,20,21].

In addition to these anti-VEGF medications, intravitreal corticosteroids like dexamethasone (DEX) or fluocinolone acetonide implants are usually used as a second-line strategy in this pathological condition. In particular, it has been shown that the sustained-release intravitreal 0.7 mg corticosteroid DEX implant (Ozurdex^®^, AbbVie Inc., North Chicago, IL, USA) is beneficial in treating RVO [22,23]. It comprises a biodegradable capsule composed of glycolytic and lactic acid polymers. Furthermore, it has been shown that an intravitreal DEX implant can reduce inflammation by inhibiting several inflammatory cytokines, hence reducing edema, fibrin deposition, inflammatory cell migration, and capillary leakage [24]. However, the possibility of using the intravitreal DEX implant as a first line of treatment or making a switch from anti-VEGF therapy in case of refractory macular edema must be highly considered to guarantee maximum visual recovery and the least retinal damage to the patient.

For this reason, the aim of this review is to primarily analyze this retinal vascular disease, focusing on its pathophysiology and on the clinical and ocular imaging characteristics that may favor a switch to intravitreal DEX implant therapy from previous anti-VEGF treatment, also through the scientific evidence derived from published clinical studies.

## 2. Risk Factors of RVO

Probably the strongest independent risk factor for all forms of RVO is systemic hypertension [25,26], particularly in the older (over 50) age group. In fact, this population frequently has uncontrolled or recently diagnosed hypertension, and when hypertension is not well managed, RVO in the same or contralateral eye is known to reoccur. Moreover, compared to controls, hyperlipidemia was shown to be twice as frequent in RVO patients (both CRVO and BRVO) [26,27]. Both systemic hypertension and hyperlipidemia could determine an increased risk of RVO because they favor a narrowing of the caliber of the retinal vessels and an increase in their tortuosity [26].

Concerning diabetes mellitus, its association with RVO is weaker than systemic hypertension and hyperlipidemia and has not been found to be consistent across all studies [25,26], with a potential stronger association with CRVO than with BRVO [25,26].

Though rare, myeloproliferative disorders may cause elevated blood viscosity, which is known to be linked to CRVO. Similar to this, certain uncommon systemic inflammatory diseases that induce systemic vasculitis (such as polyarteritis nodosa and Behçet’s disease) can also produce retinal vasculitis, which can result in RVO, particularly in younger patients [28]. In this case, the underlying systemic disease and its treatment are directly related to the origin and management of the RVO.

Recently, a lot of attention has been paid to the possible contribution of thrombophilia to the development of RVO and, specifically, CRVO [29]. Thrombophilia is a hematological condition that can be acquired (such as antiphospholipid syndrome) or congenital (such as Factor V Leiden; hyperhomocysteinemia; and protein C, protein S, and antithrombin deficiencies), which may be more significant in the younger age groups [29,30]. However, clinical studies varied in their ability to demonstrate a meaningful correlation between RVO and protein C, protein S, and antithrombin III insufficiency and factor V Leiden/activated protein C resistance [29]. Conversely, phospholipid-specific antibodies trigger the coagulation cascade in the antiphospholipid syndrome, resulting in venous and arterial thrombosis. The anticardiolipin antibody assay can be used to identify this antibody, or a lupus anticoagulant test can be used to measure its impact on coagulation [30]. Ocular symptoms might appear in up to 8% of patients affected by antiphospholipid syndrome, and there is a strong correlation between this syndrome and CRVO [29]. Nonetheless, to ascertain the degree of correlation between antiphospholipid syndrome and RVO, further studies are needed. Hyperhomocysteinemia, which increases the risk of venous and arterial thrombosis, has several origins, including uncommon enzyme abnormalities that result in homocystinuria [29]. Numerous investigations have cast doubt on the necessity of performing comprehensive testing for thrombophilia in RVO patients in the absence of a medical history that may be indicative. Nonetheless, their findings have demonstrated a strong enough correlation between hyperhomocysteinemia and CRVO to advocate for the advantages of testing for this condition, which may be treated with folic acid and vitamins B6 and B12 supplementations [31,32]. For this reason, based on the available data, it would be advisable to save general thrombophilia screening for elderly patients with a history of thromboembolic events and for younger patients without any additional general risk factors, rather than recommending it for all patients with RVO [29].

In addition, as systemic conditions, cigarette smoking and renal disease have also been associated with RVO [3].

**Figure 1:**
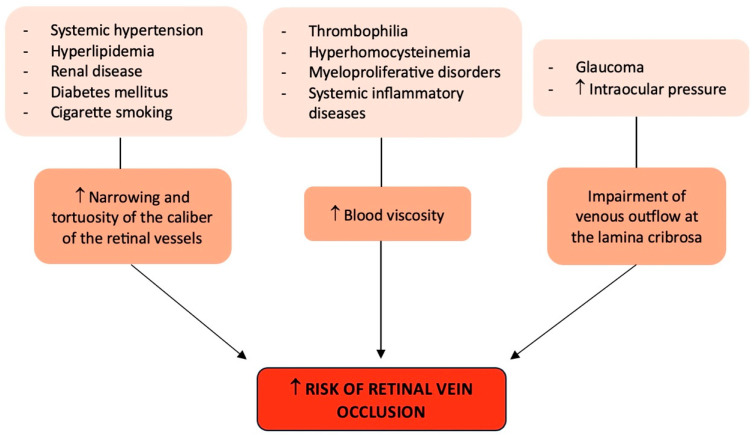
Graphical summary of the risk factors involved in retinal vein occlusion. ↑: increase.

Finally, glaucoma and high intraocular pressure are two ocular risk factors for CRVO that may impair venous outflow at the lamina cribrosa [3] (Figure 1).

## 3. Pathophysiology of RVO

As is well known, the most common cause of vision loss in RVO is the development of macular edema [1,9]. For this reason, gaining a basic grasp of the pathophysiology behind macular edema might help to comprehend the mechanism of action of the drugs that have been recommended for RVO.

As previously mentioned, the thrombosis of a retinal vein can partially impede blood flow both from the eye and within the vein. Starling’s law states that if the ensuing increase in intraluminal pressure is large enough, blood components will transude into the retina. Thus, interstitial fluid and proteins in the retina will rise as a result. The latter will prolong tissue edema and raise interstitial oncotic pressure, which will obstruct capillary perfusion, thus resulting in ischemia. There is no all-or-none distinction when it comes to retinal ischemia, since people who are declared non-ischemic might instead have varied degrees of this condition [33].

Moreover, it is commonly known that inflammation has an impact on the course and result of vitreoretinal diseases, such as RVO [32]. In fact, when compared to healthy controls, vitreous levels of the soluble cytokines such as VEGF, monocyte chemoattractant protein-1, and interleukin (IL) 6 and 8 were significantly higher in RVO, and particularly in CRVO [34,35].

Though the precise way in which these elements interact is still up for debate, our knowledge of the role of VEGF is growing. It is brought on by tissue hypoxia, such as retinal ischemia, and it operates on endothelial-cell-membrane-bound receptors with tyrosine kinase activity as an angiogenic and vasopermeable agent [16]. In particular, the degree of retinal ischemia and the intensity of macular edema were both linked with the levels of VEGF and IL-6. The rapid retinal ischemia that happens in CRVO is probably going to cause overproduction of VEGF, which can be produced by many types of ocular tissue, including Muller cells, endothelial cells, and retinal pigment epithelial cells [34]. According to Starling’s law, which was previously discussed, the increased vascular permeability caused by VEGF will probably contribute to the macular edema. Theoretically, even if the primary venous obstruction is removed, macular edema may continue for a much longer time because of a self-reinforcing cycle mediated by the VEGF itself.

**Figure 2:**
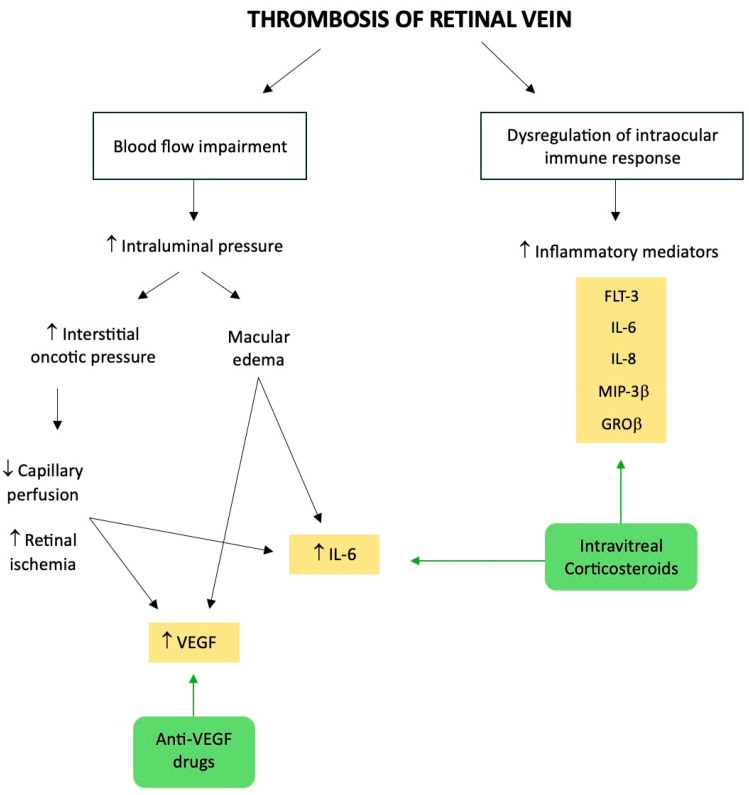
Illustrative summary of the pathophysiology of retinal vein occlusion, highlighting the molecular targets of the intravitreal treatments. ↑: increase; ↓: decrease.

In conclusion, in addition to VEGF, it is crucial to highlight the important role of inflammation in case of RVO. In fact, it has been demonstrated that RVO patients had significantly higher intraocular levels of inflammatory mediators, in particular Flt-3 L, IL-8, MIP-3β, and GROβ, which were all more pronounced in CRVO than in BRVO [36]. These results may suggest that retinal findings in RVO may be driven primarily by a dysregulation of intraocular immune response [36], pointing out how the inflammation may play a key role in macular edema secondary to RVO (Figure 2).

## 4. Clinical and Imaging Parameters in RVO

**Figure 3:**
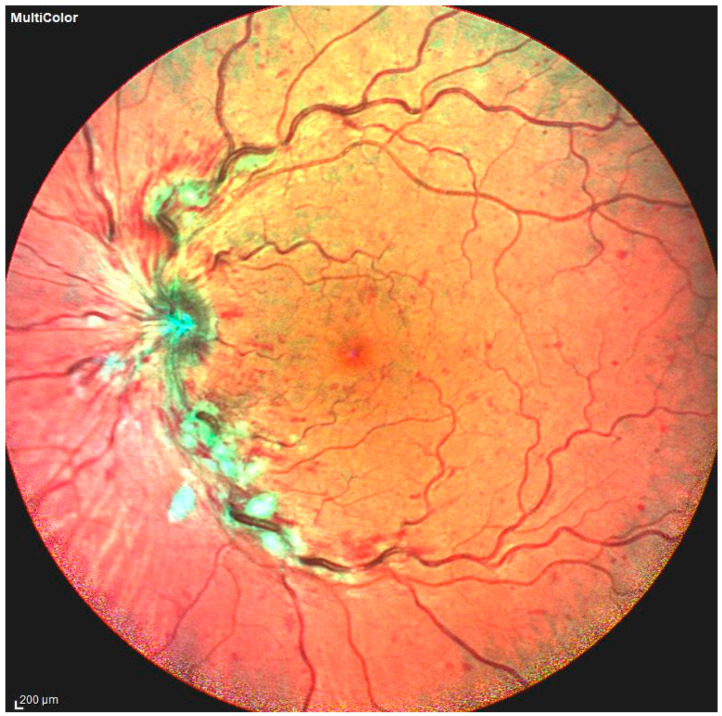
Photo fundus of central retinal vein occlusion, with some of the main features of this retinal condition: vascular tortuosity increase, venous dilatation, and retinal and intraretinal hemorrhages.

RVO manifests clinically as increased vascular tortuosity, venous dilatation, cotton-wool spots, macular edema, and retinal and intraretinal hemorrhages [37] (Figure 3).

Hard exudates, microaneurysms, vein sclerosis, vascular shunts at the optic disc, artery constriction and sheathing, vitreous hemorrhage, tractional retinal detachment, and neovascularization of the iris, retina, or optic disc are other late characteristics that may also be present [38]. Typically, thrombi can be seen downstream of arteriovenous crossing; however, it is not evident if the thrombus is the cause or effect of RVO [39]. At fundus fluorescein angiography, the distinctive findings are the delayed filling of the occluded retinal vein and capillary nonperfusion, with the intraretinal hemorrhages resulting in dye blockage. Moreover, late leakage may occur due to macular edema or retinal neovascularization.

Optical coherence tomography (OCT) has been extensively utilized in RVO to evaluate the presence of cotton-wool spots, hard exudates, macular edema, and subretinal fluid (SRF) [40,41]. In particular, several clinical studies have indicated that even if SRF is frequently seen at presentation, baseline SRF does not consistently indicate long-term functional or anatomic outcomes [42,43]. Furthermore, although with a lower clinical significance than diabetic macular edema, hyperreflective foci are considered OCT biomarkers of inflammation also in RVO, particularly in BRVO [44,45]. In addition, they are associated with worse visual outcomes, most likely due to damage to the photoreceptor layer [46].

**Figure 4:**
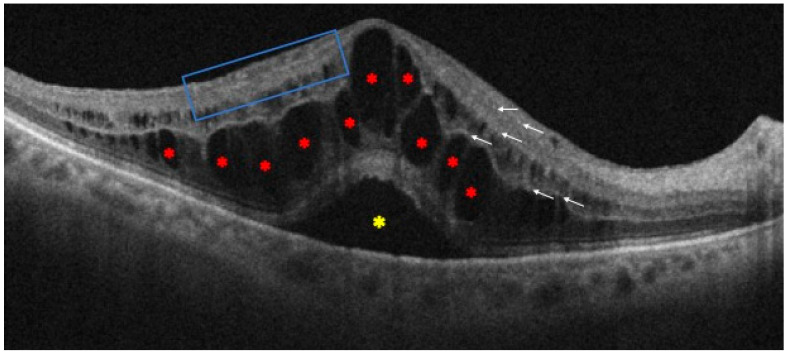
Optical coherence tomography scan of patient with central retinal vein occlusion. It is possible to observe some of the main morphological biomarkers: intraretinal cysts (red asterisks), subretinal fluid (yellow asterisk), some hyperreflective foci (white arrows), and disorganization of the retinal inner layers (blue box).

Moreover, evaluating the degree and duration of macular edema is crucial since improved outcomes in RVO have been linked to a shorter duration [47], together with the integrity of the connection between the inner and outer segments of the photoreceptors, which is essential to the visual prognosis [48,49,50]. In addition, thicker central subfield thickness, changes to the outer plexiform layer, and a higher percentage of external limiting membrane rupture at three months are all OCT features linked to refractory macular edema [51]. However, confounding factors that may alter connection with visual acuity include macular ischemia, atrophy, and hemorrhages [52]. Furthermore, the disorganization of retinal inner layers (DRIL) is another significant biomarker, whose morphological extent has been linked to the degree of vision loss, indicating the level of cell destruction that goes along with macular edema. In fact, an early recovery within three months is a critical element affecting the visual acuity outcomes after a year, as measured by both DRIL and ellipsoid zone disruption [53] (Figure 4).

The examination of the superficial capillary plexus (SCP) and deep capillary plexus (DCP) in RVO has been made possible by clinical studies using OCT angiography [54,55], with both the SCP and DCP of RVO eyes showing an increase in the foveal avascular zone [56,57]. Since SCP is closer to the retinal arterioles and has fewer connections, areas of nonperfusion are more common in DCP [58]. Furthermore, cystoid pockets—black circular regions devoid of a flow signal—are easier to spot with OCT angiography than fundus fluorescein angiography or OCT and have a bigger effect on both plexuses [59]. Recently, collaterals have been demonstrated in the DCP and SCP of RVO eyes [60,61,62]. These vessels form in the optic disc or retina during 6–24 months as a result of hemodynamic overload and rearrangement [63]. Deep capillaries and intraretinal connections that are difficult to detect with fundus fluorescein angiography and dilated fundoscopy can be found using OCT angiography [64]. These vessels are claimed to be more common in eyes with significant BRVO or the ischemic type than in macular BRVO or the non-ischemic type, notwithstanding disagreements about the genesis and course of these vessels in the setting of macular edema [61]. However, a recent study found that collateral vessels correlated positively with a larger foveal avascular zone and with central subfield thickness and negatively with visual acuity [64].

Finally, the presence of macular edema in RVO may correlate with a decreased choriocapillaris flow density and with an increased choroidal vascularity index [65,66].

All the discussed OCT and OCT angiography biomarkers in case of RVO are summarized in Table 1.

## 5. Therapeutic Options for RVO

Strong evidence that patients with RVO had higher intraocular VEGF levels than the control group [67,68] supports the use of anti-VEGF molecules in treating macular edema secondary to RVO. For this reason, intravitreal injections of anti-VEGF drugs are currently the gold standard of care for macular edema in RVO due to their demonstrated efficacy in addressing the underlying pathophysiology of this disease [69]. In fact, the VEGF pathway is activated in response to hypoxia caused by RVO, determining neovascularization and increasing retinal vascular permeability, thus provoking edema [70]. The two current approved anti-VEGF agents for treating the macular edema secondary to the RVO are ranibizumab and aflibercept [71].

By phospholipase A2 inhibition, corticosteroids impede the arachidonic acid pathway and have an anti-inflammatory effect, reducing the production of mediators of inflammation, such as thromboxanes, leukotrienes, and prostaglandins [72]. Additionally, they also reduce the concentrations of chemokines and cytokines, including macrophage chemoattractant protein-1, interleukin-1, and interleukin-17, which are all implicated in the development of macular edema in RVO [72]. The stabilization of the tight connections between retinal capillary cells can lower vascular permeability and fluid buildup in the retina, which supports their use in case of macular edema related to RVO. Furthermore, their pleiotropic activity includes neuroprotection and VEGF suppression [45]. For this reason, corticosteroids could be considered second-line medications, particularly when anti-VEGF treatment fails to provide the desired results even after a loading dosage. However, they should be used as first-line treatment in patients who have just suffered a major cardiovascular event or are unable to attend monthly checkups for the anti-VEGF administration, in addition to patients showing OCT biomarkers of inflammation [69,73].

Prior to the development of intravitreal treatments, the most effective method for treating BRVO-related macular edema was focal laser photocoagulation. With BRVO and vision decreased to 20/40 or worse due to macular edema, the macular grid laser greatly improved visual acuity in the BRVO eyes, as shown by the branch vein occlusion study [74]. On the other hand, the central vein occlusion study found that using a macular grid laser in eyes with CRVO did not enhance visual acuity [75]. Currently, the laser photocoagulation is considered the gold standard only for the management of RVO-related complications, such as retinal neovascularization in the areas of retinal nonperfusion, thus reducing the risk of iris neovascularization and vitreous hemorrhages [74,75].

Lastly, the subthreshold micropulse laser can induce a biological reaction without endangering the retina by utilizing a variety of laser wavelengths [76]. In particular, the yellow subthreshold micropulse laser has been explored as a possible therapeutic option for diabetic macular edema and RVO, thus lowering the financial burden of repeated anti-VEGF injections and maintaining or improving morpho-functional outcomes [76,77].

## 6. Switching to Intravitreal DEX Implant in Case of RVO

Although therapy with anti-VEGF drugs remains the gold standard for the treatment of RVO, it must also be considered that some RVO patients may not respond adequately to this therapy and, therefore, need to switch to corticosteroid therapy as soon as possible to ensure the best visual, anatomical, and functional recovery. For this reason, several clinical studies have investigated the timing for this switch and the clinical and imaging parameters useful for making this therapeutic choice.

Chiquet and colleagues [78] compared the visual and anatomical outcomes after treatment with DEX or anti-VEGF injections in 102 naïve patients with macular edema secondary to RVO. After 1 month, the frequency of “good responders”, defined as central macular thickness (CMT) less than or equal to 250 μm in time-domain OCT or 300 μm in spectral-domain OCT, was significantly lower in the anti-VEGF group than in the DEX group (11% vs. 28%). At 3 months, both groups showed significant improvements in best corrected visual acuity (BCVA) and reduction in CMT. The DEX group had a more substantial increase in BCVA compared to the anti-VEGF group, although the decrease in CMT was similar for both groups. Notably, a higher proportion of patients in the anti-VEGF group (31% vs. 13%) switched treatments by the third month due to insufficient therapeutic response or the occurrence of side effects. Conversely, no significant differences in visual or anatomical outcomes were observed at 6 and 12 months in patients who remained on their initial treatment.

The same study group subsequently examined BCVA and CMT changes in patients with macular edema due to RVO after switching from bevacizumab (3 intravitreal monthly injections) to an intravitreal DEX implant (AD group) and vice versa (1 DEX implant before switch, DA group) [79]. The switch was made if CMT remained above 300 μm and/or visual acuity improvement was less than 5 ETDRS letters after optimal treatment with the same drug or in cases of early recurrence. Forty-eight eyes, 40 in the AD group and 8 in the DA group, were assessed. In the AD group, BCVA improved significantly at 1 month, while the DA group showed no significant improvement in BCVA. The AD group also experienced significant CMT reduction at 1, 6, and 12 months. In contrast, the DA group showed no BCVA changes at any time point and only a significant CMT reduction at 1 month. Therefore, the AD switching strategy appears to have better efficacy than DA.

The efficacy of the switch from anti-VEGF to DEX implant was also evaluated by Wolfe et al. [80], who divided 23 patients into non-responders (<20% decrease in CMT) or responders (edema improved but switch for longer treatment duration). After switching to a DEX implant following a minimum of 2 anti-VEGF treatments, only non-responders showed significant improvements in visual acuity, but both groups exhibited significant improvement in CMT.

The LOUVRE study [81], a 24-month, prospective, multicenter, longitudinal, observational study carried out in France, also evaluated the intravitreal steroid treatment switch. Particularly, 375 consecutive patients with macular edema following RVO were treated with the DEX implant. The study demonstrated significant improvements in BCVA at 6 and 24 months, regardless of whether the patients were treatment-naïve or had received previous treatments. Similar to the findings by Chiquet et al. [79], patients who switched from DEX implants to other RVO treatments did not show improved outcomes.

An Israelian retrospective case series [82] evaluated 10 patients who were switched from bevacizumab, after at least 3 anti-VEGF injections, to the DEX implant, reporting a significative improvement in visual acuity and CMT. Despite a significative raise in the intraocular pressure, no intraocular pressure measurement exceeded 21 mmHg one year after the switch. Moreover, comparing these results with other switched therapies, the number of injections was significative lower in DEX-switched eyes.

Pielen et al. [83] compared anatomical and functional outcomes in patients with RVO and refractory macular edema who switched from anti-VEGF therapy to DEX implant, those who switched from DEX implant to anti-VEGF therapy, and treatment-naïve patients. The most common reasons for a switch were stagnation of BCVA and/or CMT due to chronic macular edema. Among the three groups, although naïve eyes gained 10 ETDRS letters, no group showed a significative BCVA improvement. Consistent with the previous studies [79,81], CMT remained persistently higher at the end of follow-up in the DEX–anti-VEGF group, while it significantly improved in anti-VEGF–DEX group and in naïve patients.

In the SCORE2 study [84], the anti-VEGF–DEX switch presented different outcomes. In particular, switching therapy was administered in eyes with macular edema secondary to central retinal or hemiretinal vein occlusion that had a poor response to bevacizumab or aflibercept after 6 months. The patients were switched to aflibercept or a DEX implant, respectively. Only the group switched to aflibercept showed a significant improvement in visual acuity and CMT; however, it is worth noting that the study lacked a control group and only 14 patients were switched to the DEX implant.

The use of the DEX implant as a rescue therapy was also investigated by Yap and co-authors [85]. Specifically, they administered a single DEX implant in 62 patients who showed an unsatisfactory clinical and anatomical response to at least 6 consecutive anti-VEGF injections. One month after the switch, a significant gain in mean visual acuity was obtained. Moreover, the DEX implant significantly improved visual acuity and CMT compared to preceding anti-VEGF at 1, 2, and 3 months. However, a significant elevation in intraocular pressure was observed at 30 and 60 days with 31% of CRVO and 11% of BRVO patients experiencing an intraocular pressure ≥25 mmHg.

These outcomes were also consistent with a recent systematic review and meta-analysis published by Yuan et al. including four previous studies [86,87,88,89,90]. Indeed, it reported that in patients with macular edema resistant to anti-VEGF, a single DEX implant provided BCVA improvement and CMT reduction with an efficacy lasting 6 months and without serious adverse events in any of the included studies [86].

In a retrospective multicenter study, Houben et al. [91] assessed the effect of DEX implant as a switch therapy in persistent macular edema secondary to RVO in vitrectomized and non-vitrectomized eyes. Overall, all the patients had a sustained significant CMT reduction throughout the study. By the end of follow-up, BCVA had only improved in vitrectomized eyes. However, multivariable regression analyses showed no significant association between vitrectomy status and CMT or BCVA change after the first and last injection [91].

A rescue therapy with a DEX implant was also evaluated in a recent Chinese study focused on long-term outcomes of anti-VEGF treatment with 5+PRN regimen for macular edema due to CRVO [92]. In this setting, the DEX implant was implemented in nine patients, resulting in improved CMT in all treated patients.

Finally, another interesting insight on DEX implant switch therapy was reported in a recent Turkish study, which compared the outcomes of early or late switch from anti-VEGF injections to DEX implant in treatment-naïve patients with macular edema secondary to BRVO [93]. After the loading dose, the patients were divided into two groups: early DEX group (34 eyes who received the DEX implant after 3 loading doses) and late DEX group (34 eyes who started the DEX implant after 6 months). The late switch showed to be more effective than the early switch at the end of the first year. In particular, the late switch group presented a higher number of patients gaining ≥15 ETDRS letters and a higher anatomical improvement than the early switch group.

All the discussed studies are summarized in Table 2.

## 7. Key Points for a Timely Switch

This review aims to analyze the available scientific literature regarding the clinical and imaging criteria for switching from intravitreal anti-VEGF therapy to an intravitreal DEX implant in patients affected by macular edema secondary to RVO, also focusing on OCT parameters that may guide this therapeutical choice or identify naive patients in whom corticosteroid therapy could represent the first-line therapy.

In fact, RVO represents the second most common cause of retinal vascular disease-related visual loss, and for this reason, identifying the best possible therapy for each patient would be crucial to guarantee the maximum possible anatomical and functional recovery, minimizing the harmful effects of this retinal disease.

All clinical studies regarding the switch from anti-VEGF therapy to DEX implant focused on the number of performed intravitreal anti-VEGF injections, BCVA, and CMT as the main clinical and imaging parameters to rely on for this choice, with some differences among the studies [78,79,80,81,82,83,84,85,86,87,88,89,90,91,92,93] (Table 2). Overall, a CMT greater than 300 µm, or a worsening of BCVA, or a BCVA improvement less than 5 ETDRS letters after at least 3 anti-VEGF injections are all considered favorable parameters for a switch to DEX implant therapy.

Unfortunately, this review is unable to provide precise indications on the exact timing for the switch to DEX implant in RVO patients. Indeed, the available clinical data are very variable, and specific imaging parameters that can help in this therapeutic choice have not yet been identified.

However, an important limitation of all these clinical studies could be that they did not consider all those OCT biomarkers of inflammation that have been demonstrated to be positively correlated with a good response to corticosteroid therapy, even for a first-line treatment, such as subretinal fluid and hyperreflective foci [40,41,42,43,44,45,46,47,48,49,50,51,52,53].

In fact, focusing on these OCT biomarkers could better guide not only the choice of initial therapy but also the right timing to switch therapy started with anti-VEGF, which, to date, represents the gold standard for the RVO treatment [69], with also the possibility of reducing the number of intravitreal injections the patient undergoes.

In terms of safety, intravitreal DEX has a minor risk of systemic and local side effects compared to other steroids (fluocinolone, triamcinolone). In fact, the risk of systemic adverse effects is lowered by its little systemic absorption and intravitreal administration method. In addition, DEX has a high solubility in water and leaves the eye more quickly than other steroids, which may lower the frequency of local events. However, differently from anti-VEGF drugs, side effects such as cataract advancement and elevated intraocular pressure might result from intravitreal steroid attraction for lens and trabecular meshwork cells [94]. However, only a few patients need filtration surgery, since the intraocular pressure increase is often controlled with topical reducing medications [95]. Considering the formation of cataracts, it is preferable to avoid and postpone DEX implant in patients who are phakic, even if there is still debate over the increased incidence of this complication in patients receiving the DEX implant compared to anti-VEGF therapy [94,96,97].

Moreover, we must not overlook the fact that the DEX implant, both in RVO and diabetic macular edema, represents the first-line treatment in all those patients who may not be suitable for repeated anti-VEGF treatments, such as patients with high-risk cardiovascular disease or who are unable to attend monthly or frequent appointments, with poor compliance, severe macular edema (more than 500 microns), history of cataract surgery, and scheduled for cataract surgery [94,98,99].

In addition, the DEX implant is also preferred by cost-benefit analyses, considering that it has already been demonstrated that an improvement in macular edema can be achieved with a lower number of DEX implants compared to the use of anti-VEGF drugs, thus resulting in lower costs [94,100,101,102,103].

Finally, another important aspect to take into consideration is the recurrence rate and rebound of symptoms in RVO. In fact, a rebound macular edema effect can occur typically after 3–4 months, but it was demonstrated to not affect functional or anatomical recovery when retreatment with the DEX implant is provided [104]. Similar findings were also found for anti-VEGF therapy, where foveal thickness fluctuations could longitudinally impair the visual acuity and foveal photoreceptor status during the observation period, thus influencing the final outcomes [105].

## 8. Conclusions

Based on the current literature, the switch from anti-VEGF therapy to DEX implant therapy in RVO is mainly guided by BCVA and CMT, generally after a minimum of three injections, without considering the OCT biomarkers of inflammation that would allow us to optimally target patients who would benefit more from DEX implant.

Therefore, it would be recommendable in the future to carry out further clinical studies on RVO in which these OCT biomarkers could also be considered to evaluate the best timing for the switch from anti-VEGF therapy to intravitreal corticosteroids, to guarantee the best visual outcome and to reduce the number of intravitreal injections, with a better cost–benefit ratio and a reduction in the hospital burden.

## Figures and Tables

**Table 1:** Main optical coherence tomography and optical coherence tomography angiography biomarkers in case of retinal vein occlusion.

Biomarker.	Significance
**Optical** **c** **oherence tomography**	
Intraretinal cysts	Demonstrate the presence of macular edema, especially related to vasogenic effects
Central subfield thickness	Increased thickness correlates positively with worse visual outcomes
Disorganization of retinal inner layers	Its morphological extent is linked to the degree of vision loss, indicating the level of cell destruction
External limiting membrane rupture	Associated with worse visual outcomes
Ellipsoid zone disruption	Associated with worse visual outcomes
Hyperreflective foci	Inflammation biomarker associated with worse visual outcomes, most likely due to damage to the photoreceptor layer
Subretinal fluid	Inflammation biomarker frequently seen at presentation, but it does not consistently indicate long-term functional or anatomic outcomes
**Optical coherence tomography angiography**	
Foveal avascular zone	Increased both in superficial capillary plexus and in deep capillary plexus
Collateral vessels	Demonstrated both in superficial capillary plexus and in deep capillary plexus, correlating positively with larger foveal avascular zone and with central subfield thickness and negatively with visual acuity
Choriocapillaris flow density	Decreased in case of macular edema secondary to retinal vein occlusion
Choroidal vascularity index	Increased in case of macular edema secondary to retinal vein occlusion

**Table 2:** Summary of the studies analyzing the switch from anti-VEGF therapy to DEX implant in RVO.

Author (Year)	Ref.	Population	Number of Anti-VEGF Injections before Switch	Reason to Switch	Outcomes
Chiquet et al. (2015)	[78]	One hundred two naïve patients (64 in the anti-VEGF group, 38 in the DEX group) were included and evaluated at baseline and 1, 3, 6, and 12 months after the treatment. Patients were defined as “good responders” if CMT was less than or equal to 250 μm in time-domain OCT or 300 μm in spectral-domain OCT after the injections. After 3 months, 25 patients switched treatment, i.e., changed from anti-VEGF to DEX or vice versa.	Three monthly injections (bevacizumab 1.25 mg or ranibizumab 0.5 mg)	Insufficient therapeutic response or the occurrence of side effects	At month 3, BCVA had increased significantly in both groups, with a higher increase in the DEX group; furthermore, CMT decreased significantly in both groups. A higher number of patients in the anti-VEGF group changed the treatment compared to the DEX group (31% vs. 13%).
Chiquet et al. (2016)	[79]	48 eyes, 40 in the anti-VEGF DEX sequence (AD group), 8 in the DEX anti-VEGF sequence (DA group)—were included and evaluated at baseline, 1, 3, 6 and 12 months after the switch. Patients treated first with anti-VEGF received three bevacizumab injections within 3 months. Patients treated first with DEX received one DEX implant for a minimum period of 4 months and were then switched to bevacizumab.	Three monthly bevacizumab 1.25 mg	The switch was decided if CMT remained >300 μm and/or if BCVA improvement was less than 5 EDTRS letters after optimal treatment with the same drug or in case of early recurrence.	In the AD group, BCVA significantly improved at 1 month, and CMT decreased significantly at 1, 6, and 12 months. In contrast, the DA group showed no change in BCVA at any time point, and CMT decreased only at 1 month but not at subsequent evaluations.
Wolfe et al. (2016)	[80]	23 eyes previously treated with a minimum of 2 anti-VEGF treatments were included and switched to DEX implant. 14 patients were non-responders and 9 responders to anti-VEGF	Minimum of 2 anti-VEGF injections (drugs not specified)	The switch was based on poor response (persistent macular edema, <20% decrease in CMT at the time of switch) or on the need to reduce frequent intravitreal anti-VEGF injections.	Non-responders showed significant improvements in visual acuity after the switch. Both groups exhibited significant improvement in CMT after the switch. A reduction in CMT by ≤25% one month after one anti-VEGF injection is predictive of poor response to anti-VEGF treatment.
Korobelnik et al. (2016)	[81]	375 eyes with macular edema at baseline were treated with DEX, 153 patients were treatment naïve and 239 had received previous treatment.	Only the drugs were specified (bevacizumab 1.25 mg and ranibizumab 0.5 mg)	Consecutive patients, all patients were treated with DEX regardless to previous treatment	BCVA improved significantly from baseline at 6 months in patients regardless of their treatment status at baseline. Patients who switched from DEX implant to other RVO treatments did not have improved outcomes.
Hanhart et al. (2017)	[82]	37 patients with macular edema secondary to RVO were included, 10 eyes were switched to DEX implant.	At least 3 monthly bevacizumab 1,25 mg injections	Patients were switched if CMT persisted at ≥300 μm, if there was less than a 20% improvement in cystoid macular edema from baseline, and/or if BCVA improvement was less than 1 Snellen line after anti-VEGF treatment.	Eyes switched to DEX exhibited at 12 months a significative improvement in BCVA and CMT with a lower number of injections in comparison with other switched options.
Pielen et al. (2017)	[83]	47 eyes were divided in three groups: patients treated with anti-VEGF and successively with DEX implant; patients treated with DEX and then switched to anti-VEGF; naïve patients.	At least 3 intravitreal anti-VEGF injections (drugs were not specified)	The switch was based on clinical findings. Patients were classified as poor responder or no responder either according functional (BCVA) or morphological parameters (CMT). Patients were switched for BCVA deterioration, BCVA stagnation, for patient’s choice, for intraocular pressure decompensation, or for unknown reason	BCVA not significant statistically improved in all 3 groups. Median CMT decrease was most pronounced in treatment naïve patients compared to anti-VEGF refractory eyes and dexamethasone-refractory eyes.
Ip et al. (2018)	[84]	49 eyes with central-RVO- or hemiretinal-RVO-associated macular edema and poor response to aflibercept (14 eyes) or bevacizumab (35 eyes) treatment at month 6 were switched to DEX implant or to aflibercept, respectively.	Only the drugs (aflibercept and bevacizumab) were clearly specified.	A poor or marginal response at month 6 was defined as (1) BCVA <20/80 or BCVA improvement of fewer than 5 letters from baseline, and (2) spectral-domain OCT with CMT 300 μm or greater, presence of intraretinal cystoid spaces, or subretinal fluid	Eyes treated with aflibercept after a poor response to bevacizumab had improvement in BCVA and CMT. Few eyes had a poor response to aflibercept, and therefore, few eyes were switched to dexamethasone, with no statistical significative improvement in BCVA or CMT.
Yap et al. (2021)	[85]	Sixty-two injections in 62 patients associated with 26% central RVO and 74% branch RVO were treated with one DEX implant after a poor response.	At least 6 intravitreal anti-VEGF injections (bevacizumab, ranibizumab, aflibercept and combinations)	Anti-VEGF failure criteria included six consecutive anti-VEGF injections with a final response ≤5 ETDRS letters, CMT reduction ≤20%, and a documented unsatisfactory clinical response	DEX represents a useful rescue therapy in cases of anti-VEGF failure for macular edema following RVO, resulting in improved functional and anatomical outcomes at 30 days. A significant elevation of intraocular pressure was observed, with a peak rise of 10 mmHg or greater at 60 days.
Yuan et al. (2022)	[86]	99 eyes presenting macular edema secondary to branch RVO in 72 eyes, central RVO in 26 eyes, and hemiretinal RVO in one eye underwent one intravitreal DEX implant and were followed up for 6 months after presenting anti-VEGF therapy resistance.	The mean number of anti-VEGF injections (bevacizumab, ranibizumab, aflibercept) was 3.83–9	Clinical and anatomical absent response to a mean number of anti-VEGF injections ranging from 3.83 to 9.	RVO patients with refractory macular edema benefited significantly from switching therapy to DEX implant, with efficacy lasting 6 months after a single-dose application. In addition, DEX implant presented a good safety profile with only minor adverse events.
Alshahrani et al. (2016)	[87]	53 eyes with refractory macular edema secondary to central RVO (13 eyes), branch RVO (14 eyes), and diabetic macular edema (26 eyes) were treated with a single 0.7 mg DEX implant.	At least 6 monthly anti-VEGF agents including bevacizumab, ranibizumab and aflibercept	Refractory macular edema was defined as no improvement of 2 or more lines in Snellen BCVA and of the CMT on spectral-domain OCT that remained above 350 μm.	BCVA improved significantly at 1 month and 3 months. The CMT decreased significantly at 1, 3, and 6 months. Fourteen (26%) eyes developed high intraocular pressure after DEX implant, and it was successfully controlled with topical medications, and cataract progressed in one (1.8%) eye.
Manousaridis et al. (2017)	[88]	Eleven eyes with ranibizumab-resistant macular edema secondary to RVO were treated with a single DEX implant.	The mean number of ranibizumab injections was 9 (range 6–16)	Macular edema was considered refractory to ranibizumab if no change in the pattern of macular fluid on OCT and no change in BCVA.	Treatment with DEX resulted in improvement of BCVA and reduction of CMT in patients with ranibizumab refractory macular edema due to RVO at 3 months. However, BCVA gain did not last up to 6 months. Therefore, a re-injection before this time point could be considered.
Lee et al. (2017)	[89]	38 eyes with macular edema for branch RVO that did not respond to at least 2 consecutive bevacizumab intravitreal injections were switched to one DEX implant.	Two or more monthly bevacizumab injections	Patients refractory to 2 or more intravitreal bevacizumab were switched if presented no improvement or worsening BCVA, <150 µm reduction in CMT, and CMT >300 µm.	A single DEX implant improved both functional and anatomical outcomes for up to 6 months in about half of the patients with macular edema secondary to branch RVO who are refractory to intravitreal bevacizumab, particularly in those with initially low baseline BCVA.
Georgalas et al. (2019)	[90]	23 eyes, 13 branch RVO, and 10 central RVO patients with persistent macular edema (>250 μm) after at least five anti-VEGF injections were switched to repeated intravitreal DEX administrated on an “as-needed” protocol.	At least five anti-VEGF injections (ranibizumab or aflibercept)	Persistent macular edema (>250 μm)	DEX implant represented an effective and safe alternative in patients with branch RVO and central RVO who have failed anti-VEGF therapy. In the branch RVO group, the mean CMT and BCVA significantly improved at 6 and 12 months. In the central RVO group, neither the mean CMT nor BCVA improved significantly at 6 months. At 12 months, CMT was significantly improved, but BCVA lacked significant improvement. Cataract progression was a rare event (2/23 eyes), while transient steroid-induced ocular hypertension (5/23 eyes) was managed successfully with intraocular-pressure-lowering medication.
Houben et al. (2018)	[91]	72 eyes (40 with central RVO and 32 with branch RVO) were examined and received at least one DEX implant. 31 patients had prior vitrectomy.	Only the drugs (ranibizumab and/or bevacizumab) were clearly specified	Refractory macular edema was defined as CMT >290 µm despite intravitreal anti-VEGF agents	Multiple DEX implants are effective in reducing CMT in patients resistant to previous treatments and appear to be similarly effective in vitrectomized and non-vitrectomized eyes. 16.7% patients suffered from an intraocular pressure greater than 25 mmHg, which was well controlled with intraocular-pressure-lowering medication in 83.3% of cases.
Ye et al. (2023)	[92]	27 eyes with macular edema associated with non-ischemic central RVO (15 patients) and ischemic central RVO (12 patients) were treated with anti-VEGF agents, followed by reinjections as needed or pro-re-nata regimen. Retinal laser photocoagulation or DEX implant were implemented in both groups when necessary.	Five consecutive intravitreal injections of conbercept or ranibizumab, followed by reinjections as needed or pro-re-nata regimen.	Drug switching was applied to central RVO-macular edema patients who showed persistent or recurrent macular edema, despite repeated anti-VEGF therapy.	BCVA improved significantly in all patients. Both the non-ischemic central RVO and the ischemic central RVO groups achieved BCVA improvement compared to the baseline at all visit points. The mean CMT was statistically reduced compared to baseline at all visit points in all the eyes. DEX was applied to four eyes in the non-ischemic central RVO group and five eyes in the ischemic central RVO group.
Yozgat et al. (2024)	[93]	Sixty-eight treatment-naïve branch RVO patients who started anti-VEGF treatment were included. After the loading dose, the patients were divided into two groups: early DEX group (n:34) (DEX implant started after 3 loading doses) and late DEX group (n:34) (DEX treatment started after 6 months).	A loading dose of bevacizumab (number of injections not clearly specified).	Insufficient therapeutic response	The late switch showed to be more effective than early switch. Specifically, the late DEX group presented a higher number of patients gaining more than 15 letters and a higher anatomical improvement than the early DEX group (26 vs. 14 eyes; 136.9 µm vs. 115.3 µm).

VEGF: vascular endothelial growth factor; DEX: dexamethasone; CMT: central macular thickness; OCT: optical coherence tomography; BCVA: best corrected visual acuity; RVO: retinal vein occlusion.

## References

[1] Scott I.U., Campochiaro P.A., Newman N.J., Biousse V. (2020). Retinal vascular occlusions. Lancet.

[2] Klein R., Klein B.E., Moss S.E., Meuer S.M. (2000). The epidemiology of retinal vein occlusion: The Beaver Dam Eye Study. Trans. Am. Ophthalmol. Soc..

[3] Mitchell P., Smith W., Chang A. (1996). Prevalence and Associations of Retinal Vein Occlusion in Australia. The Blue Mountains Eye Study. Arch. Ophthalmol..

[4] Klein R., Moss S.E., Meuer S.M., Klein B.E. (2008). The 15-year cumulative incidence of retinal vein occlusion: The Beaver Dam Eye Study. Arch Ophthalmol..

[5] Rogers S., McIntosh R.L., Cheung N., Lim L., Wang J.J., Mitchell P., Kowalski J.W., Nguyen H., Wong T.Y., International Eye Disease Consortium (2010). The Prevalence of Retinal Vein Occlusion: Pooled Data from Population Studies from the United States, Europe, Asia, and Australia. Ophthalmology.

[6] Vitiello L., Salerno G., Coppola A., Abbinante G., Gagliardi V., Pellegrino A. (2023). Simultaneous Branch Retinal Artery and Central Retinal Vein Occlusion Improved with No Ocular Therapy: A Case Report. Tomography.

[7] Hayreh S.S. (2005). Prevalent misconceptions about acute retinal vascular occlusive disorders. Prog. Retin. Eye Res..

[8] Hayreh S.S. (2021). Photocoagulation for retinal vein occlusion. Prog. Retin. Eye Res..

[9] Romano F., Lamanna F., Gabrielle P.H., Teo K.Y., Parodi M.B., Iacono P., Fraser-Bell S., Cornish E.E., Nassisi M., Viola F. (2023). Update on Retinal Vein Occlusion. Asia-Pac. J. Ophthalmol..

[10] Etheridge T., Blodi B., Oden N., Van Veldhuisen P., Scott I.U., Ip M.S., Mititelu M., Domalpally A. (2021). Spectral Domain OCT Predictors of Visual Acuity in the Study of COmparative Treatments for REtinal Vein Occlusion 2: SCORE 2 Report 15. Ophthalmol. Retina.

[11] Ong C.J.T., Wong M.Y.Z., Cheong K.X., Zhao J., Teo K.Y.C., Tan T.-E. (2023). Optical Coherence Tomography Angiography in Retinal Vascular Disorders. Diagnostics.

[12] Castro-Navarro V., Monferrer-Adsuara C., Navarro-Palop C., Montero-Hernández J., Cervera-Taulet E. (2022). Optical coherence tomography biomarkers in patients with macular edema secondary to retinal vein occlusion treated with dexamethasone implant. BMC Ophthalmol..

[13] Kazantzis D., Sergentanis T.N., Machairoudia G., Dimitriou E., Kroupis C., Theodossiadis G., Theodossiadis P., Chatziralli I. (2023). Correlation Between Imaging Morphological Findings and Laboratory Biomarkers in Patients with Retinal Vein Occlusion. Ophthalmol. Ther..

[14] McIntosh R.L., Rogers S.L., Lim L., Cheung N., Wang J.J., Mitchell P., Kowalski J.W., Nguyen H.P., Wong T.Y. (2010). Natural History of Central Retinal Vein Occlusion: An Evidence-Based Systematic Review. Ophthalmology.

[15] Rogers S.L., McIntosh R.L., Lim L., Mitchell P., Cheung N., Kowalski J.W., Nguyen H.P., Wang J.J., Wong T.Y. (2010). Natural History of Branch Retinal Vein Occlusion: An Evidence-Based Systematic Review. Ophthalmology.

[16] Hang A., Feldman S., Amin A.P., Ochoa J.A.R., Park S.S. (2023). Intravitreal Anti-Vascular Endothelial Growth Factor Therapies for Retinal Disorders. Pharmaceuticals.

[17] Karia N. (2010). Retinal vein occlusion: Pathophysiology and treatment options. Clin. Ophthalmol..

[18] Holz F.G., Roider J., Ogura Y., Korobelnik J.-F., Simader C., Groetzbach G., Vitti R., Berliner A.J., Hiemeyer F., Beckmann K. (2013). VEGF Trap-Eye for macular oedema secondary to central retinal vein occlusion: 6-month results of the phase III GALILEO study. Br. J. Ophthalmol..

[19] Boyer D., Heier J., Brown D.M., Clark W.L., Vitti R., Berliner A.J., Groetzbach G., Zeitz O., Sandbrink R., Zhu X. (2012). Vascular endothelial growth factor Trap-Eye for macular edema secondary to central retinal vein occlusion: Six-month results of the phase 3 COPERNICUS study. Ophthalmology.

[20] Campochiaro P.A., Heier J.S., Feiner L., Gray S., Saroj N., Rundle A.C., Murahashi W.Y., Rubio R.G., BRAVO Investigators (2010). Ranibizumab for macular edema following branch retinal vein occlusion: Six-month primary end point results of a phase III study. Ophthalmology.

[21] Brown D.M., Campochiaro P.A., Singh R.P., Li Z., Gray S., Saroj N., Rundle A.C., Rubio R.G., Murahashi W.Y., CRUISE Investigators (2010). Ranibizumab for macular edema following central retinal vein occlusion: Six-month primary end point results of a phase III study. Ophthalmology.

[22] Iovino C., Mastropasqua R., Lupidi M., Bacherini D., Pellegrini M., Bernabei F., Borrelli E., Sacconi R., Carnevali A., D’Aloisio R. (2020). Intravitreal Dexame-thasone Implant as a Sustained Release Drug Delivery Device for the Treatment of Ocular Diseases: A Comprehensive Review of the Literature. Pharmaceutics.

[23] Coscas G., Augustin A., Bandello F., de Smet M.D., Lanzetta P., Staurenghi G., Parravano M.C., Udaondo P., Moisseiev E., Soubrane G. (2014). Retreatment with Ozurdex for Macular Edema Secondary to Retinal Vein Occlusion. Eur. J. Ophthalmol..

[24] Nicula C., Nicula D., Rednik A., Bulboaca A., Crișan O. (2020). Morphological and Functional Outcomes after Intravitreal Dexamethasone Injection for Macular Edema in Patients with Central Vein Occlusion at 48-Week Follow-Up. J. Ophthalmol..

[25] Koizumi H., Ferrara D.C., Bruè C., Spaide R.F. (2007). Central Retinal Vein Occlusion Case-Control Study. Am. J. Ophthalmol..

[26] O’mahoney P.R.A., Wong D.T., Ray J.G. (2008). Retinal Vein Occlusion and Traditional Risk Factors for Atherosclerosis. Arch. Ophthalmol..

[27] Cheung N., Klein R., Wang J.J., Cotch M.F., Islam A.F.M., Klein B.E.K., Cushman M., Wong T.Y. (2008). Traditional and Novel Cardiovascular Risk Factors for Retinal Vein Occlusion: The Multiethnic Study of Atherosclerosis. Investig. Opthalmol. Vis. Sci..

[28] Terao R., Fujino R., Ahmed T. (2022). Risk Factors and Treatment Strategy for Retinal Vascular Occlusive Diseases. J. Clin. Med..

[29] Fegan C.D. (2002). Central retinal vein occlusion and thrombophilia. Eye.

[30] Marongiu F., Ruberto M.F., Marongiu S., Mameli A., Barcellona D. (2024). Do we need more guidance on thrombophilia testing? Challenges and special considerations. Expert Rev. Hematol..

[31] Turello M., Pasca S., Daminato R., Dello Russo P., Giacomello R., Venturelli U., Barillari G. (2010). Retinal vein occlusion: Evaluation of “classic“ and “emerging“ risk factors and treatment. J. Thromb. Thrombolysis.

[32] Lendzioszek M., Mrugacz M., Bryl A., Poppe E., Zorena K. (2023). Prevention and Treatment of Retinal Vein Occlusion: The Role of Diet—A Review. Nutrients.

[33] Campochiaro P.A., Hafiz G., Shah S.M., Nguyen Q.D., Ying H., Do D.V., Quinlan E., Zimmer-Galler I., Haller J.A., Solomon S.D. (2008). Ranibizumab for Macular Edema Due to Retinal Vein Occlusions; Implication of VEGF as a Critical Stimulator. Mol. Ther..

[34] Yoshimura T., Sonoda K.H., Sugahara M., Mochizuki Y., Enaida H., Oshima Y., Ueno A., Hata Y., Yoshida H., Ishibashi T. (2009). Comprehensive analysis of inflammatory immune mediators in vitreoretinal diseases. PLoS ONE.

[35] Funk M., Kriechbaum K., Prager F., Benesch T., Georgopoulos M., Zlabinger G.J., Schmidt-Erfurth U. (2009). Intraocular Concentrations of Growth Factors and Cytokines in Retinal Vein Occlusion and the Effect of Therapy with Bevacizumab. Investig. Opthalmol. Vis. Sci..

[36] Zhou Y., Qi J., Liu H., Liang S., Guo T., Chen J., Pan W., Tan H., Wang J., Xu H. (2023). Increased intraocular inflammation in retinal vein occlusion is independent of circulating immune mediators and is involved in retinal oedema. Front. Neurosci..

[37] Wong T.Y., Scott I.U. (2010). Clinical practice. Retinal-vein occlusion. N. Engl. J. Med..

[38] Cochran M.L., Mahabadi N., Czyz C.N. (2024). Branch Retinal Vein Occlusion. StatPearls.

[39] Muraoka Y., Tsujikawa A., Murakami T., Ogino K., Kumagai K., Miyamoto K., Uji A., Yoshimura N. (2013). Morphologic and Functional Changes in Retinal Vessels Associated with Branch Retinal Vein Occlusion. Ophthalmology.

[40] Murakami T., Tsujikawa A., Ohta M., Miyamoto K., Kita M., Watanabe D., Takagi H., Yoshimura N. (2007). Photoreceptor status after re-solved macular edema in branch retinal vein occlusion treated with tissue plasminogen activator. Am. J. Ophthalmol..

[41] Voo I., Mavrofrides E.C., Puliafito C. (2004). Clinical applications of optical coherence tomography for the diagnosis and management of macular diseases. Ophthalmol. Clin. N. Am..

[42] Segal O., Yavnieli R., Mimouni M., Rabina G., Geffen N., Moisseiev E., Nemet A.Y. (2021). Optical Coherence Tomography Biomarkers Predicting Visual Acuity Change after Intravitreal Bevacizumab Injections for Macular Edema Secondary to Branch Retinal Vein Occlusion. Ophthalmologica.

[43] Hoeh A.E., Ach T., Schaal K.B., Scheuerle A.F., Dithmar S. (2009). Long-term follow-up of OCT-guided bevacizumab treatment of macular edema due to retinal vein occlusion. Graefe’s Arch. Clin. Exp. Ophthalmol..

[44] Tao Y., Ge L., Su N., Li M., Fan W., Jiang L., Yuan S., Chen Q. (2024). Exploration on OCT biomarker candidate related to macular edema caused by diabetic retinopathy and retinal vein occlusion in SD-OCT images. Sci. Rep..

[45] Vitiello L., Salerno G., Coppola A., De Pascale I., Abbinante G., Gagliardi V., Lixi F., Pellegrino A., Giannaccare G. (2024). Switching to an Intravitreal Dexamethasone Implant after Intravitreal Anti-VEGF Therapy for Diabetic Macular Edema: A Review. Life.

[46] Chatziralli I.P., Sergentanis T.N., Sivaprasad S. (2016). Hyperreflective Foci as an Independent Visual Outcome Pre-Dictor in Macular Edema due to Retinal Vascular Diseases Treated with Intravitreal Dexame-Thasone or Ranibizumab. Retina.

[47] Scott I.U., VanVeldhuisen P.C., Oden N.L., Ip M.S., Blodi B.A., Hartnett M.E., Cohen G., Standard Care versus Corticosteroid for Retinal Vein Occlusion Study Investigator Group (2011). Baseline Predictors of Visual Acuity and Retinal Thickness Outcomes in Patients with Retinal Vein Occlusion: Standard Care versus Corticosteroid for Retinal Vein Occlusion Study Report 10. Ophthalmology.

[48] Kang H.M., Chung E.J., Kim Y.M., Koh H.J. (2013). Spectral-domain optical coherence tomography (SD-OCT) patterns and response to intravitreal bevacizumab therapy in macular edema associated with branch retinal vein occlusion. Graefe’s Arch. Clin. Exp. Ophthalmol..

[49] Mejía M.E., Ríos H.A., Rosenstiehl S., Rodríguez F.J. (2023). Optical coherence tomography angiography as predictor of visual outcomes in retinal vein occlusion treated with antiangiogenic therapy. Eur. J. Ophthalmol..

[50] Ota M., Tsujikawa A., Murakami T., Kita M., Miyamoto K., Sakamoto A., Yamaike N., Yoshimura N. (2007). Association between integrity of foveal photoreceptor layer and visual acuity in branch retinal vein occlusion. Br. J. Ophthalmol..

[51] Moon B.G., Cho A.R., Kim Y.N., Kim J.G. (2018). Predictors of Refractory Macular Edema after Branch Retinal Vein Occlusion following Intravitreal Bevacizumab. Retina.

[52] Gregori N.Z., Rattan G.H., Rosenfeld P.J., Puliafito C.A., Feuer W., Flynn H.W., Berrocal A.M., Al-Attar L., Dubovy S., Smiddy W.E. (2009). Safety and Efficacy of Intravitreal Bevacizumab (Avastin) for the Management of Branch and Hemiretinal Vein Occlusion. Retina.

[53] Chan E.W., Eldeeb M., Sun V., Thomas D., Omar A., Kapusta M.A., Galic I.J., Chen J.C. (2019). Disorganization of Retinal Inner Layers and Ellipsoid Zone Disruption Predict Visual Outcomes in Central Retinal Vein Occlusion. Ophthalmol. Retina.

[54] Ben Abdesslem N., Haddar S., Mahjoub A., Mahjoub A., Ghorbel M., Mahjoub H., Knani L., Krifa F. (2021). Retinal vein occlusions: An OCT- Angiography analysis. Tunis Med..

[55] Stone L.G., Grinton M.E., Talks J.S. (2021). Delayed follow-up of medical retina patients due to COVID-19: Impact on disease activity and visual acuity. Graefe’s Arch. Clin. Exp. Ophthalmol..

[56] Samara W.A., Shahlaee A., Sridhar J., Khan M.A., Ho A.C., Hsu J. (2016). Quantitative Optical Coherence Tomography Angiography Fea-tures and Visual Function in Eyes with Branch Retinal Vein Occlusion. Am. J. Ophthalmol..

[57] Suzuki N., Hirano Y., Tomiyasu T., Esaki Y., Uemura A., Yasukawa T., Yoshida M., Ogura Y. (2016). Retinal Hemodynamics Seen on Optical Coherence Tomography Angiography Before and After Treatment of Retinal Vein Occlusion. Investig. Opthalmol. Vis. Sci..

[58] Martinet V., Guigui B., Glacet-Bernard A., Zourdani A., Coscas G., Soubrane G., Souied E.H. (2012). Macular edema in central retinal vein occlusion: Correlation between optical coherence tomography, angiography and visual acuity. Int. Ophthalmol..

[59] Glacet-Bernard A., Sellam A., Coscas F., Coscas G., Souied E.H. (2016). Optical Coherence Tomography Angiography in Retinal Vein Occlusion Treated with Dexamethasone Implant: A New Test for Follow-Up Evaluation. Eur. J. Ophthalmol..

[60] Iida Y., Muraoka Y., Ooto S., Suzuma K., Murakami T., Iida-Miwa Y., Ghashut R., Tsujikawa A. (2017). Morphologic and Functional Retinal Vessel Changes in Branch Retinal Vein Occlusion: An Optical Coherence Tomography Angiography Study. Am. J. Ophthalmol..

[61] Suzuki N., Hirano Y., Tomiyasu T., Kurobe R., Yasuda Y., Esaki Y., Yasukawa T., Yoshida M., Ogura Y. (2019). Collateral vessels on optical coherence tomography angiography in eyes with branch retinal vein occlusion. Br. J. Ophthalmol..

[62] Tsuboi K., Sasajima H., Kamei M. (2019). Collateral Vessels in Branch Retinal Vein Occlusion: Anatomic and Functional Analyses by OCT Angiography. Ophthalmol. Retina.

[63] Im C.Y., Lee S.Y., Kwon O.W. (2002). Collateral vessels in branch retinal vein occlusion. Korean J. Ophthalmol..

[64] Arrigo A., Aragona E., Lattanzio R., Scalia G., Bandello F., Parodi M.B. (2021). Collateral Vessel Development in Central and Branch Retinal Vein Occlusions Are Associated with Worse Visual and Anatomic Outcomes. Investig. Opthalmol. Vis. Sci..

[65] Okamoto M., Yamashita M., Sakamoto T., Ogata N. (2018). Choroidal Blood Flow and Thickness as Predictors for Response to Anti-Vascular Endothelial Growth Factor Therapy in Macular Edema Secondary TO Branch Retinal Vein Occlusion. Retina.

[66] Aribas Y.K., Hondur A.M., Tezel T.H. (2020). Choroidal vascularity index and choriocapillary changes in retinal vein occlusions. Graefe’s Arch. Clin. Exp. Ophthalmol..

[67] Pe’er J., Folberg R., Itin A., Gnessin H., Hemo I., Keshet E. (1998). Vascular endothelial growth factor upregulation in human central retinal vein occlusion. Ophthalmology.

[68] Noma H., Funatsu H., Yamasaki M., Tsukamoto H., Mimura T., Sone T., Jian K., Sakamoto I., Nakano K., Yamashita H. (2005). Pathogenesis of Macular Edema with Branch Retinal Vein Occlusion and Intraocular Levels of Vascular Endothelial Growth Factor and Interleukin-6. Arch. Ophthalmol..

[69] Schmidt-Erfurth U., Garcia-Arumi J., Gerendas B.S., Midena E., Sivaprasad S., Tadayoni R., Wolf S., Loewenstein A. (2019). Guidelines for the Management of Retinal Vein Occlusion by the European Society of Retina Specialists (EURETINA). Ophthalmologica.

[70] Daruich A., Matet A., Moulin A., Kowalczuk L., Nicolas M., Sellam A., Rothschild P.-R., Omri S., Gélizé E., Jonet L. (2018). Mechanisms of macular edema: Beyond the surface. Prog. Retin. Eye Res..

[71] Lendzioszek M., Bryl A., Poppe E., Zorena K., Mrugacz M. (2024). Retinal Vein Occlusion–Background Knowledge and Foreground Knowledge Prospects—A Review. J. Clin. Med..

[72] Rezar-Dreindl S., Eibenberger K., Pollreisz A., Bühl W., Georgopoulos M., Krall C., Dunavölgyi R., Weigert G., Kroh M., Schmidt-Erfurth U. (2016). Effect of intravitreal dexamethasone implant on intra-ocular cytokines and chemokines in eyes with retinal vein occlusion. Acta Ophthalmol..

[73] Soliman M.K., Zarranz-Ventura J., Chakravarthy U., McKibbin M., Brand C., Menon G., Cilliers H., Natha S., Ross A., Sarhan M. (2023). United Kingdom Database Study of Intravitreal Dexamethasone Implant (Ozurdex) for Macular Edema Related to Retinal Vein Occlusion. Retina.

[74] The Branch Vein Occlusion Study Group (1984). Argon Laser Photocoagulation for Macular Edema in Branch Vein Occlusion. Am. J. Ophthalmol..

[75] Natural history and clinical management of central retinal vein occlusion (1997). The Central Vein Occlusion Study Group. Arch Ophthalmol..

[76] Iovino C., Iodice C.M., Pisani D., Rosolia A., Testa F., Giannaccare G., Chhablani J., Simonelli F. (2023). Yellow Subthreshold Micropulse Laser in Retinal Diseases: An In-Depth Analysis and Review of the Literature. Ophthalmol. Ther..

[77] Grzybowski A., Sulaviková Z., Gawęcki M., Kozak I. (2024). Subthreshold laser treatment in retinal diseases: A mini review. Graefe’s Arch. Clin. Exp. Ophthalmol..

[78] Chiquet C., Dupuy C., Bron A.M., Aptel F., Straub M., Isaico R., Romanet J.P., Creuzot-Garcher C. (2015). Intravitreal dexamethasone implant versus anti-VEGF injection for treatment-naïve patients with retinal vein occlusion and macular edema: A 12-month follow-up study. Graefe’s Arch. Clin. Exp. Ophthalmol..

[79] Chiquet C., Bron A.M., Straub M., Dupuy C., Isaico R., Aptel F., Creuzot-Garcher C. (2016). Retinal Vein Occlusions: Therapeutic Switch in Macular Oedema Treatment with a 12-Month Follow-Up. Ophthalmic Res..

[80] Wolfe J.D., Shah A.R., Yonekawa Y., Al Faran A., Franklin M.S., Abbey A.M., Capone A. (2016). Receiver Operating Characteristic Curve to Predict Anti-VEGF Resistance in Retinal Vein Occlusions and Efficacy of Ozurdex. Eur. J. Ophthalmol..

[81] Korobelnik J.-F., Kodjikian L., Delcourt C., Gualino V., Leaback R., Pinchinat S., Velard M.-E. (2016). Two-year, prospective, multicenter study of the use of dexamethasone intravitreal implant for treatment of macular edema secondary to retinal vein occlusion in the clinical setting in France. Graefe’s Arch. Clin. Exp. Ophthalmol..

[82] Hanhart J., Rozenman Y. (2017). Comparison of Intravitreal Ranibizumab, Aflibercept, and Dexamethasone Implant after Bevacizumab Failure in Macular Edema Secondary to Retinal Vascular Occlusions. Ophthalmologica.

[83] Pielen A., Bühler A.D., Heinzelmann S.U., Böhringer D., Ness T., Junker B. (2017). Switch of Intravitreal Therapy for Macular Edema Sec-ondary to Retinal Vein Occlusion from Anti-VEGF to Dexamethasone Implant and Vice Versa. J. Ophthalmol..

[84] Ip M.S., Oden N.L., Scott I.U., VanVeldhuisen P.C., Blodi B.A., Ghuman T., Baker C.W., SCORE2 Investigator Group (2019). Month 12 Outcomes After Treatment Change at Month 6 Among Poor Responders to Aflibercept or Bevacizumab in Eyes with Macular Edema Secondary to Central or Hemiretinal Vein Occlusion: A Secondary Analysis of the SCORE2 Study. JAMA Ophthalmol..

[85] Yap E.T., Husein S., de Imperial-Ollero J.A.M., Colizzi B., Cordeiro M.F., Younis S. (2021). The efficacy of dexamethasone implants following anti-VEGF failure for macular oedema in retinal vein occlusion. Eur. J. Ophthalmol..

[86] Yuan Q., Gao Y., Liu Y., Xu H., Wang T., Zhang M. (2022). Efficacy of single-dose intravitreal dexamethasone implantation for retinal vein occlusion patients with refractory macular edema: A systematic review and meta-analysis. Front. Pharmacol..

[87] Alshahrani S.T., Dolz-Marco R., Gallego-Pinazo R., Diaz-Llopis M., Arevalo J.F., KKESH International Collaborative Retina Study Group (2016). Intravitreal Dexamethasone Implant for the Treatment OF Refractory Macular Edema in Retinal Vascular Diseases: Results of the KKESH International Collaborative Retina Study Group. Retina.

[88] Manousaridis K., Peter S., Mennel S. (2017). Outcome of intravitreal dexamethasone implant for the treatment of ranibizumab-resistant macular edema secondary to retinal vein occlusion. Int. Ophthalmol..

[89] Lee K.H., Kang E.C., Koh H.J. (2017). Dexamethasone Intravitreal Implant Rescue Treatment for Bevacizumab Refractory Macular Edema Secondary to Branch Retinal Vein Occlusion. Korean J. Ophthalmol..

[90] Georgalas L., Tservakis I., Kiskira E.E., Petrou P., Papaconstantinou D., Kanakis M. (2019). Efficacy and safety of dexamethasone intravitreal implant in patients with retinal vein occlusion resistant to anti-VEGF therapy: A 12-month prospective study. Cutan. Ocul. Toxicol..

[91] Houben I., De Zaeytijd J., Deghislage C., Frost N.A., Nijs I., Van Calster J. (2018). Efficacy of Multiple Dexamethasone Intravitreal Implants for Refractory Retinal Vein Occlusion-Related Macular Edema and Effect of Prior Vitrectomy. J. Ocul. Pharmacol. Ther..

[92] Ye Y., Huang Z., Wu Q.-W., Hui Y.-N., Song Y.-P. (2023). Long-term outcomes of anti-VEGF treatment with 5+PRN regimen for macular edema due to central retinal vein occlusion. Int. J. Ophthalmol..

[93] Yozgat Z., Işik M.U. (2024). Anatomical and Functional Results of Early or Late Switching from Anti-VEGF to Dexamethasone Implant in Case of Poor Anatomical Response in Naïve Patients with Macular Edema Secondary to Branch Retinal Vein Occlusion. Semin. Ophthalmol..

[94] Taloni A., Coco G., Rastelli D., Buffon G., Scorcia V., Giannaccare G. (2023). Safety and Efficacy of Dexamethasone Intravitreal Implant Given Ei-ther First-Line or Second-Line in Diabetic Macular Edema. Patient Prefer Adherence.

[95] Maturi R.K., Pollack A., Uy H.S., Varano M., Gomes A.M.V., Li X.-Y., Cui H., Lou J., Hashad Y., Whitcup S.M. (2016). Intraocular Pressure in Patients with Diabetic Macular Edema Treated with Dexamethasone Intravitreal Implant in The 3-Year Mead Study. Retina.

[96] Malclès A., Dot C., Voirin N., Agard É., Vié A.L., Bellocq D., Denis P., Kodjikian L. (2017). Real-Life Study in Diabetic Macular Edema Treated with Dexamethasone Implant: The Reldex Study. Retina.

[97] Fallico M., Lotery A., Maugeri A., Favara G., Barchitta M., Agodi A., Russo A., Longo A., Bonfiglio V., Avitabile T. (2022). Intravitreal dexamethasone implant versus anti-vascular endothelial growth factor therapy combined with cataract surgery in patients with diabetic macular oedema: A systematic review with meta-analysis. Eye.

[98] Sohn H.J., Han D.H., Kim I.T., Oh I.K., Kim K.H., Lee D.Y., Nam D.H. (2011). Changes in Aqueous Concentrations of Various Cytokines After Intravitreal Triamcinolone Versus Bevacizumab for Diabetic Macular Edema. Am. J. Ophthalmol..

[99] He Y., Ren X.J., Hu B.J., Lam W.C., Li X.R. (2018). A meta-analysis of the effect of a dexamethasone intravitreal implant versus intravitreal anti-vascular endothelial growth factor treatment for diabetic macular edema. BMC Ophthalmol..

[100] Cheng Z., Liu X. (2024). Zhi Comparing the efficacy of glucocorticoids and anti-VEGF in treating diabetic macular edema: Systematic review and comprehensive analysis. Front. Endocrinol..

[101] Khan Z., Kuriakose R.K., Khan M., Chin E.K., Almeida D.R.P. (2017). Efficacy of the Intravitreal Sustained-Release Dexamethasone Implant for Diabetic Macular Edema Refractory to Anti-Vascular Endothelial Growth Factor Therapy: Meta-Analysis and Clinical Implications. Ophthalmic Surg. Lasers Imaging Retin..

[102] Ruiz-Moreno J.M., Ruiz-Medrano J. (2023). Early-switch versus late-switch in patients with diabetic macular edema: A cost-effectiveness study. Graefe’s Arch. Clin. Exp. Ophthalmol..

[103] Lin J., Gibbons A., Smiddy W.E. (2021). Cost-Utility of Anti-Vascular Endothelial Growth Factor Treatment for Macular Edema Secondary to Central Retinal Vein Occlusion. Ophthalmol. Retin..

[104] Parodi M.B., Iacono P., De Benedetto U., Cascavilla M., Bandello F. (2012). Rebound Effect After Intravitreal Dexamethasone Implant for the Treatment of Macular Edema Secondary to Central Retinal Vein Occlusion. J. Ocul. Pharmacol. Ther..

[105] Nagasato D., Muraoka Y., Tanabe M., Nishigori N., Osaka R., Mitamura Y., Tabuchi H., Murakami T., Ooto S., Suzuma K. (2024). Foveal Thickness Fluctuations in Anti-VEGF Treatment for Central Retinal Vein Occlusion. Ophthalmol. Sci..

